# Molecular Origami: Designing Functional Molecules of the Future

**DOI:** 10.3390/molecules30020242

**Published:** 2025-01-09

**Authors:** Hitoshi Ishida, Takeshi Ito, Akinori Kuzuya

**Affiliations:** 1 Department of Chemistry and Materials Engineering, Faculty of Chemistry, Materials and Bioengineering, Kansai University, 3-3-35 Yamate-cho, Suita 564-8680, Osaka, Japan; kuzuya@kansai-u.ac.jp; 2 Graduate School of Science and Engineering, Kansai University, 3-3-35 Yamate-cho, Suita 564-8680, Osaka, Japan; t.ito@kansai-u.ac.jp; 3 Department of Mechanical Engineering, Faculty of Engineering Science, Kansai University, 3-3-35 Yamate-cho, Suita 564-8680, Osaka, Japan; 4 Organization for Research and Development of Innovative Science and Technology (ORDIST), Kansai University, 3-3-35 Yamate-cho, Suita 564-8680, Osaka, Japan

**Keywords:** molecular origami, protein origami, peptide origami, DNA origami, biosensor

## Abstract

In the field of chemical biology, DNA origami has been actively researched. This technique, which involves folding DNA strands like origami to assemble them into desired shapes, has made it possible to create complex nanometer-sized structures, marking a major breakthrough in nanotechnology. On the other hand, controlling the folding mechanisms and folded structures of proteins or shorter peptides has been challenging. However, recent advances in techniques such as protein origami, peptide origami, and de novo design peptides have made it possible to construct various nanoscale structures and create functional molecules. These approaches suggest the emergence of new molecular design principles, which can be termed “molecular origami”. In this review, we provide an overview of recent research trends in protein/peptide origami and DNA/RNA origami and explore potential future applications of molecular origami technologies in electrochemical biosensors.

## 1. Introduction

Origami is one of the most beautiful forms of art in Japanese traditional culture. This technique, which creates infinite shapes from a single sheet of paper, has been cherished by the Japanese people for centuries. Origami is an astonishing art that transforms a single sheet of paper into various shapes. Without cutting or gluing, simply by folding, a flat piece of paper can be turned into three-dimensional animals, plants, or even geometric shapes. Within this simplicity lies infinite creativity. By combining basic folding techniques, origami can also produce extremely complex shapes and designs. For example, using simple “mountain folds” and “valley folds”, one can fold the paper inward to create three-dimensional forms. The reason a single square of paper can be transformed into various shapes, such as cranes, frogs, flowers, or boxes, is due to the fundamental principle of origami being so simple yet flexible. Origami, which draws out infinite possibilities from limited materials, is highly fascinating as it is similar to the construction of functional molecules like proteins and DNA within living organisms [1].

In the field of chemical biology, the term “origami” is used most often in reference to DNA origami. DNA origami is a technique that uses DNA molecules, which carry the genetic information of living organisms, to create nanoscale structures. As the name “origami” suggests, this method involves purposefully folding a long single strand of DNA into a desired shape. Rothemund was the first to propose this technique of “folding” DNA molecules into specific shapes to create complex, nanometer-sized structures [2]. This technique became known as “DNA origami” and marked a major breakthrough in the field of nanotechnology. DNA forms stable structures by combining four types of bases (A, T, C, and G) in specific pairings. DNA origami takes advantage of this property to create a “blueprint” for the desired shape. Short, auxiliary strands of DNA are used like “staples” to fold and assemble the long strand of DNA into the correct configuration. In this way, nanoscale boxes, tubes, and even more complex three-dimensional structures can be created. The range of applications for this technology is vast. In the medical field, it has the potential to be used as a nanoscale drug delivery system. For instance, “nanorobots” created with DNA origami could be expected to deliver drugs to specific cells. Additionally, it is gaining attention as a tool for advancing biotechnology, such as in molecular-scale sensors and computer circuits.

The most important functional molecules within living organisms are proteins. Proteins are composed of 20 types of amino acids linked in long chains, which eventually fold into a three-dimensional structure, and this structure determines the protein’s function. However, predicting how a protein folds into its functional form from its amino acid sequence (primary structure) is extremely difficult. It was symbolic that the 2024 Nobel Prize in Chemistry was awarded to David Baker, who conducted pioneering research in the field of computer-assisted protein design and succeeded in creating new proteins that do not exist in nature [3]. In recent years, “Protein Origami”, which applies DNA origami techniques to proteins, has gained attention. Additionally, research on “Peptide Origami” is being conducted, which involves using metal ions or non-natural amino acids to create folded structures in peptides that do not form stable folded structures due to their short chain length. These technologies are expected to be utilized in the design of protein-based nanostructures and nanomachines, with potential applications in drug delivery systems, molecular machines that operate within cells, biomaterials for use as biocompatible materials, and even the development of artificial enzymes that do not exist in nature.

The progress of such research suggests the creation of a new class of molecules that could be called “molecular origami” in the near future. In this review, we provide an overview of “origami” research in the field of chemical biology and speculate on the “molecular origami” that could be realized in the near future. The review also delves into the field of biosensors, which is expected to be one of the most promising areas for the practical application of these new functional molecules.

## 2. Protein/Peptide Origami

Proteins act as highly functional molecules within living organisms, making the development of “artificial proteins” a much-anticipated field of research. Advances in peptide synthesis technology have made it relatively easy to synthesize proteins at the level of primary sequences. However, single chains formed by the condensation of amino acids that make up proteins undergo folding due to interactions among amino acid side chains, such as electrostatic attraction and repulsion, as well as hydrophobic interactions. As a result, their folded structures are highly influenced by factors like pH, temperature, and salt concentration. Furthermore, since there are 20 types of amino acids used in proteins, a protein composed of 100 amino acids can theoretically have 20^100^ possible combinations. Proteins in nature have refined their shapes and functions over billions of years of evolution. To create artificial proteins, designers must directly take on this process in place of evolution. Identifying combinations that yield the desired folded structure from among the vast array of possibilities is an extremely challenging task.

This section discusses the following topics: (1) Coiled coil protein origami (CCPO) using coiled coils, (2) artificial viruses that mimic the self-assembly of spherical viral capsid peptides, (3) de novo-designed metallopeptides incorporating metal ions into α-helical coiled coils, which are bundles of three α-helices, and (4) “Peptide origami”, which folds through complex formation between peptides containing metal-coordinating non-natural amino acids and metal ions.

### 2.1. Coiled Coil Protein Origami (CCPO)

**Figure 1:**
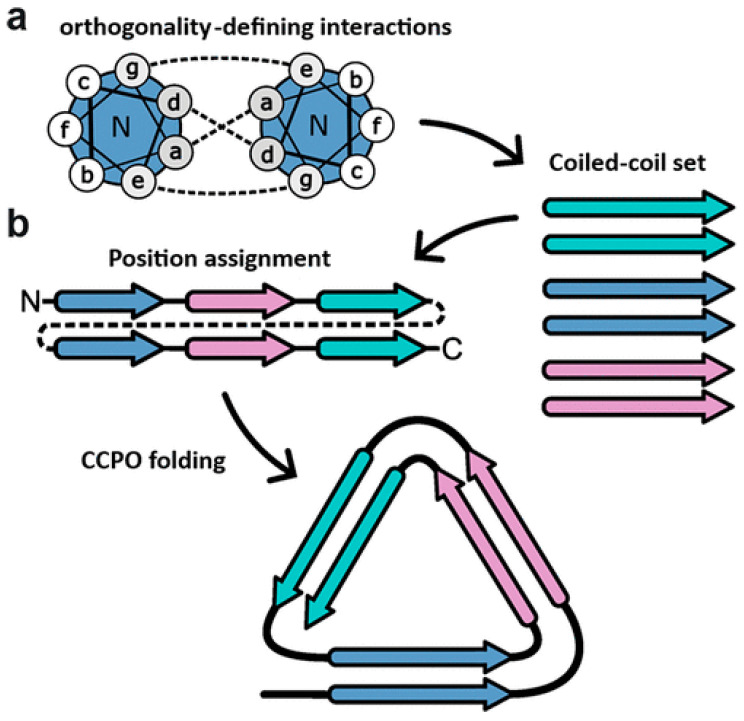
Design of triangular coiled-coil protein origami (CCPO) [4]. (**a**) Schematic representation of a parallel coiled coil between 7-residue sequences (a–g) with hydrophobic interactions between a and d residues and electrostatic interactions between e and g pairs. (**b**) Triangular CCPO was designed by sequentially arranging three pairs of parallel coiled coil building blocks.

To apply the DNA origami strategy to protein origami, sequences that bind with high specificity to particular targets are required. Coiled coil (CC) peptides are being utilized as such modular building blocks. By synthesizing proteins incorporating CC peptides, these peptides assemble into CC dimers, leading to the folding of the protein into various structures. As shown in Figure 1, an example of a protein folded into a triangular shape is presented. To date, the construction of various polyhedral structures, including tetrahedrons, square pyramids, and triangular prisms, has been reported [4,5,6,7].

Common CC peptides have a repeating structure of a 7-residue amino acid sequence, referred to as “the heptad repeat”. When this 7-residue sequence is represented as “abcdefg”, standard CC peptides typically have hydrophobic residues at positions a and d and polar residues at positions e and g. Residues a and d contribute to dimer formation through hydrophobic interactions, while residues e and g do so through electrostatic interactions. Dimers formed by CC peptides can assemble in either a parallel or antiparallel orientation. To achieve complex modular protein origami, a diverse set of CC peptides capable of binding with high specificity is required. While various CC peptides with different sizes, lengths, and orientations of constituent peptides have been developed, there is a growing need for more advanced building blocks with enhanced binding capabilities.

Coiled-coil protein origami has successfully constructed a variety of polyhedral structures [6]. In particular, the formation of large hydrophilic cavities through the folding of a single peptide chain holds promise for applications in drug delivery by utilizing these structures as molecular cages.

### 2.2. Artificial Viruses

Spherical viruses form a “capsid” through the self-assembly of proteins with a high degree of symmetry. In recent years, the development of artificial viruses that mimic the self-assembly of spherical viral capsids using peptides has been undertaken [8]. This section introduces one such example.

**Figure 2:**
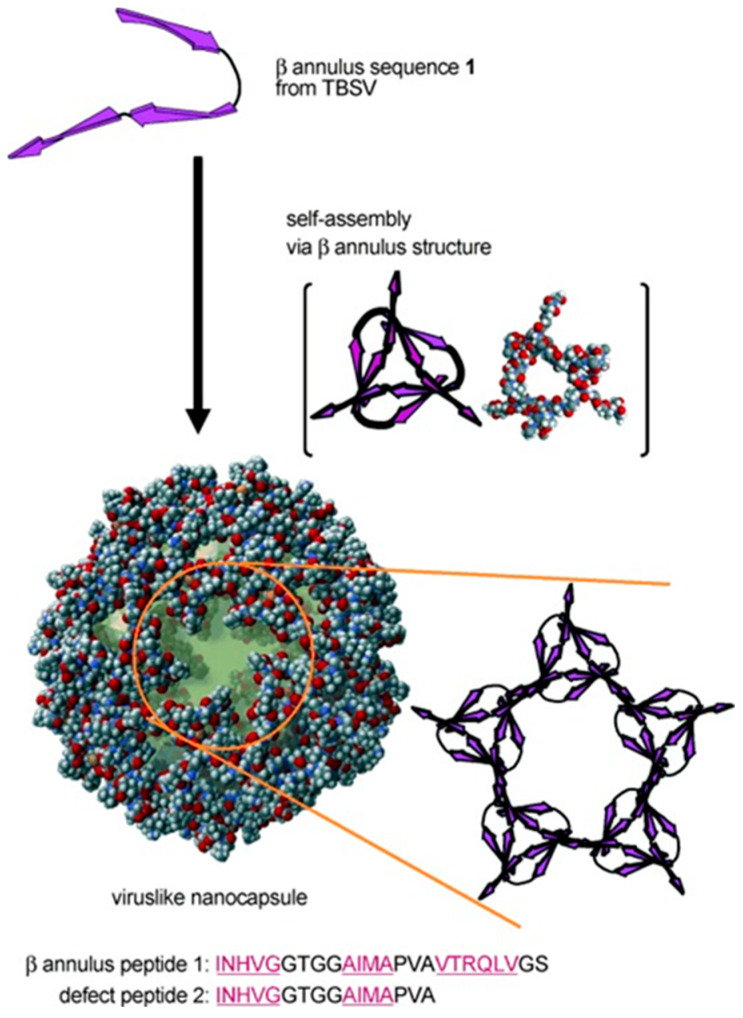
Construction of virus-like nanocapsules by self-assembly of 24-mer β-annulus peptide. The peptide forms a β-annulus trimer motif, which further assembles into a 30–50 nm artificial virus capsid. Reprinted with permission from [11]. Copyright 2010 John Wiley & Sons.

Most spherical viral capsids exhibit icosahedral symmetry. For instance, the tomato bushy stunt virus (TBSV) consists of 180 protein subunits, each containing 388 amino acid residues, and forms a spherical capsid with a diameter of approximately 33 nm [9]. Matsuura and colleagues focused on the β-annulus peptide motif (Ile69-Ser92) of the TBSV capsid, which is involved in forming the internal framework of the dodecahedron [10], and discovered for the first time that the 24-mer β-annulus peptide (amino acid sequence: INHVGGTGGAIMAPVAVTRQLVGS) self-assembles in water to form synthetic viral capsids (Figure 2) [11].

The mechanism by which protein subunits in spherical viral capsids self-assemble remains unclear in many respects. In particular, it is challenging to track self-assembly behavior in molecularly crowded environments where various biomolecules, such as proteins, nucleic acids, and lipids, are densely present. Using a fluorescently labeled β-annulus peptide as a model system, Matsuura and colleagues analyzed self-assembly behavior in crowded environments through fluorescence correlation spectroscopy (FCS) [12]. Furthermore, since natural viruses include envelope-type viruses with capsids covered by a lipid bilayer, they also succeeded in creating envelope-type artificial viral capsids that mimic this structure [13].

Artificial viruses hold promise for medical applications, such as vectors for introducing genes into cells and drug delivery systems targeting specific cells. Compared to the use of natural viruses, artificial viruses offer advantages in terms of easier risk management, such as detoxification. Additionally, due to their ability to self-assemble, artificial viruses are expected to be utilized as nanoscale devices, with potential applications in constructing nanomaterials and developing biosensors.

### 2.3. Metal-Assisted De Novo-Design Peptides

The use of de novo-designed peptides, which are peptides designed and synthesized from scratch, enables the creation of metalloproteins with various functions such as sensing, electron transfer, and catalysis by incorporating metal ions [14]. This section introduces de novo-designed metallopeptides, in which metal ions are incorporated into α-helical coiled coils composed of three *α*-helices.

**Figure 3:**
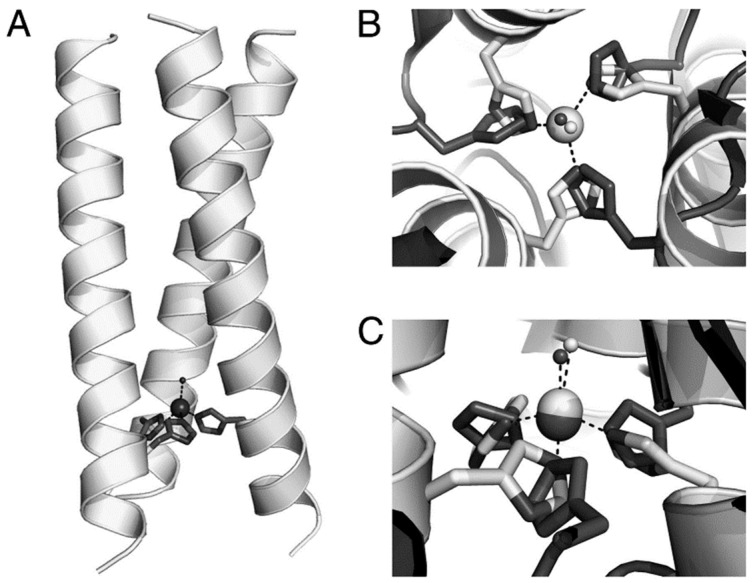
Representation of the model of the metallopeptide. (**A**) Overall structure of metallopeptide bearing Cu(His)_3_. (**B**) Comparison between Cu(His)_3_ (dark gray) and Zn(His)_3_ in the base peptide (light gray). (**C**) Side view of (**B**). Reproduced from Ref. [21].

Pecoraro and colleagues have reported various metal-containing peptides based on a 30-residue peptide, Ac-G-(LKALEEK)_4_-G-NH_2_, which forms a stable tertiary structure known as a triple-helix bundle [15,16,17,18,19,20,21]. For example, a peptide in which histidine is introduced at position 23, replacing leucine as a coordination site (L23H), forms an M(His)_3_ complex near the *C*-terminus when the peptide forms a triple-helix bundle (Figure 3). By using such a tertiary structure as a scaffold, various de novo-designed metallopeptides have been synthesized. When Zn(II) is used as the metal ion, the peptide exhibits catalytic activity for the hydrolysis of p-nitrophenyl acetate while also demonstrating carbonic anhydrase activity [18,20]. Furthermore, this catalytic activity is accelerated by substituting Zn(II) with Co(II) [15]. The Cu(His)_3_ structure is highly interesting as a redox enzyme model, but achieving it in de novo-designed peptides is generally challenging because it requires stabilization in both the reduced (Cu(I)) and oxidized (Cu(II)) states. In this system, stabilization in both states was achieved, and nitrite reductase activity was investigated [16,19,21]. Amino acid residues near the copper complex can affect the stability of the complex, even if they are not directly involved in coordination, and they also influence catalytic activity. For example, substituting lysine (K) at position 24 or glutamic acid (E) at position 27 with glutamine (Q) results in decreased activity, while substituting leucine (L) at position 19 with alanine (A) enhances activity. Furthermore, it has been reported that methylation of the δ-nitrogen of histidine controls the tautomerization and improves activity [17]. In this way, research is progressing toward the development of highly active and durable artificial metalloenzymes by examining the effects of amino acid substitutions in the peptide chain.

### 2.4. Artificial Metalloproteins

2,2′-bipyridine (bpy) binds to various metal ions to form stable metal complexes. By utilizing this property, the introduction of bipyridyl ligands into peptides can induce the association of metal ions with the peptides. For example, Koide et al. reported that introducing bipyridyl ligands at the *N*-terminus of collagen peptides and reacting them with iron(II) ions forms collagen triple helices [22]. Chmielewski et al. demonstrated that introducing bipyridyl ligands into the side chains of collagen peptides induces the formation of collagen peptide fibers [23] and disk-shaped fibers [24].

Un-natural α-amino acids with bipyridine in their side chains were developed by Imperiali, and peptides containing these amino acids have been synthesized [25,26]. Imperiali et al. also synthesized 5′-amino-2,2′-bipyridine-5-carboxylic acid (5Bpy), which contains a bipyridyl group in the amino acid backbone [27]. This unnatural amino acid, an asymmetric bipyridyl derivative, was initially synthesized via heterocoupling of two different pyridyl precursors. However, a method was later reported that starts with a symmetric bipyridyl derivative and utilizes differences in solubility between asymmetric intermediates [28], followed by further improvements to this method [29].

**Figure 4:**
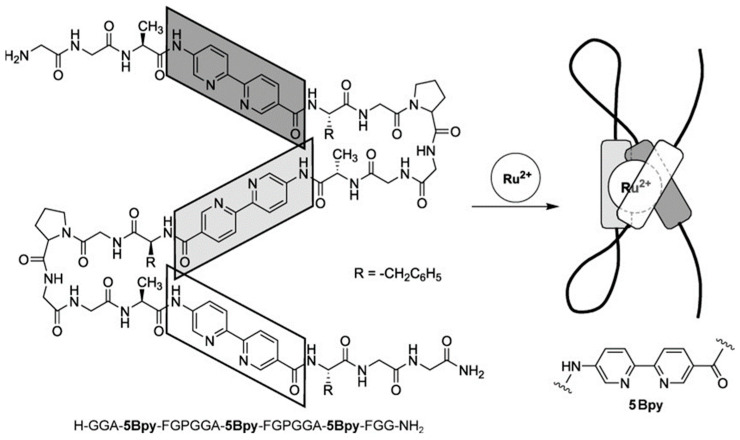
Molecular design of the artificial metalloprotein with a ruthenium(II) tris(bipyridyl) complex as the core. Three 5Bpy residues inserted in the backbone fold the peptide through the formation of a ruthenium tris(bipyridine) complex. Reprinted with permission from [30]. Copyright 2006 John Wiley & Sons.

Ishida et al. synthesized a 21-mer peptide containing three units of the bipyridine-type un-natural amino acid 5Bpy and reported that the peptide folds through the formation of a ruthenium tris(bipyridine) complex (Figure 4) [30]. Two types of folded structures were identified, each with optical isomers (Δ/Λ), resulting in four different isomers. These isomers were isolated, and their absorption and CD spectra, as well as emission spectra characteristic of ruthenium complexes (room-temperature phosphorescence), were reported. This artificial metalloprotein, which does not rely on the peptide’s secondary structure, achieves folding with a very short peptide chain and exhibits a folding structure completely different from that of de novo-designed peptides. It has been named “peptide origami”.

López et al. synthesized a peptide chain with Pro introduced between two units of the unnatural amino acid 5Bpy and reported its Co(II), Ni(II), and Zn(II) complexes [31]. They found that peptides containing two units of this unnatural bipyridyl amino acid bind strongly to these metal ions, and the folding structure is controlled by the chirality of the Pro residue in the linker region. Furthermore, they reported that in artificial metalloproteins formed through the coordination of Fe(II) with peptides containing three units of 5Bpy, the folding structure is also regulated by the chirality of the Pro residue introduced into the peptide chain connecting the 5Bpy units [32]. This technique holds promise for expanding the potential of artificial metalloproteins, enabling the control of three-dimensional structures solely through metal ions.

**Figure 5:**
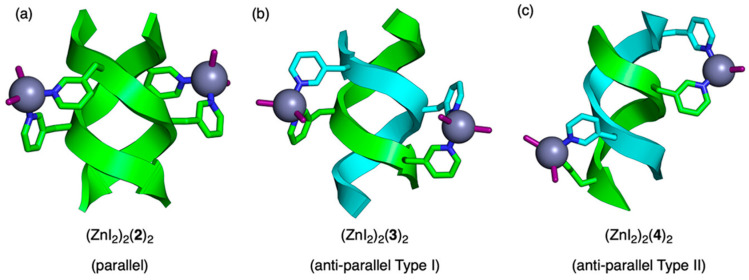
Structures of Zn(II) complexes of (**a**) parallel double-stranded (ds)-β-helix, (**b**) type I antiparallel ds-β-helix, and (**c**) type II antiparallel ds-β-helix [33].

In recent years, a unique molecular design of artificial metalloproteins that combines peptide secondary structures with coordination to a metal ion has been reported [33]. Peptides composed of alternating sequences of L- and D-amino acids are known to form double-stranded β-helices. Sawada et al. synthesized a peptide in which two residues of Val in an octapeptide composed of alternating L- and D-Val were replaced with β-(3-pyridyl)-L-alanine. These peptide chains form both parallel and antiparallel double-stranded (ds) β-helices, which are stabilized by the complexation of the introduced pyridylalanine side chains with Zn(II) ions (Figure 5). This artificial metalloprotein can achieve a stable three-dimensional structure with a very short peptide chain. In the future, the development of functional molecules based on this structure as a core is highly anticipated.

## 3. DNA Origami

**Figure 6:**
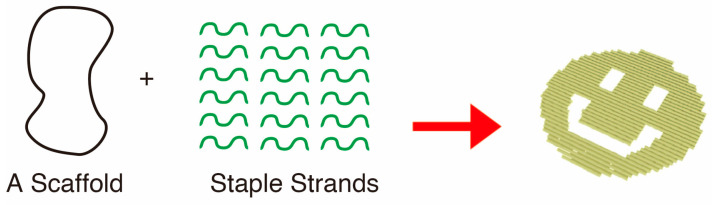
DNA origami. Long single-stranded scaffold strands are folded into any desired 2D (or 3D) nanostructures such as "smiley face" in the right with the aid of a large number (~220) of short complementary strands called “staple strands” under annealing between 80–25 °C (red arrow).

Since its introduction in 2006 as the second-generation technique to construct DNA nanostructures, DNA origami has now become the core technology in the field of structural DNA nanotechnology (Figure 6) [2,34]. This provides sophisticated and precise nanostructures by folding long single-stranded DNA called scaffold strand (usually 7249-nt circular single-stranded M13mp18 phage genome is used) with many short complementary strands called staple strands (typically more than 200 strands of approximately 32 bases are used) in a predesigned arrangement. Initially, only simple two- or three-dimensional structures were created [35], but after extensive studies and developments, even complicated three-dimensional assemblies of multiple modules up to several tens of microns can be prepared today [36]. Not only for the preparation of diverse nanostructures, but also DNA origami has been used as scaffolds to arrange metallic nanomaterials [37]. Nanomechanical molecular devices are another attractive application of the technique [38]. Recent trends also include the construction of DNA origami nanopores [39] and excellent applications as DDS carriers studied mainly by researchers in China [40].

### 3.1. DNA Origami as Nanoelectric Matrial

**Figure 7:**
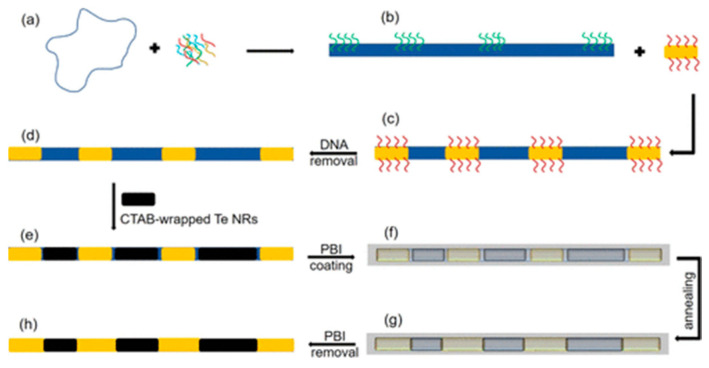
Au-Te-Au junction formation on rod-shaped DNA origami by annealing. DNA origami template (left in (**b**)) was folded from M13mp18 DNA (left in (**a**)) with staple strands (right in (**a**)). DNA-modified Au nanorods (right in (**b**)) were bound via DNA hybridization (**c**) and after DNA was removed using gold plating solution ((**d**)), cetyltrimethylammonium bromide (CTAB)-coated Te nanorods were then bound in the gaps through electrostatic interaction (**e**). Au-Te connections were formed after annealing of polybenzimidazole (PBI)-coated complex (**f**–**h**). Reprinted with permission from [44]. Copyright 2021 American Chemical Society.

The composites of metallic nanomaterials and DNA origami were mainly intended for applications in the field of optics, such as plasmonic materials [41]. Reports on the construction of nanosized conductive materials, in contrast, have increased in recent years. Vigorous research is particularly being conducted by Woolley’s group [42,43,44]. The first work was reported in 2018 [42]. They fabricated Au nanowires ∼130 nm long and 10 nm in diameter by arranging 25–30 nm long Au nanorods in a C- or X-shape on a rectangular DNA origami and connecting them by anisotropic growth. Probe electrodes were then fabricated by electron beam-induced metal deposition (EBID), and the conductivity of Au nanowires was measured by a four-point measurement technique, yielding a resistance of 4.24 × 10^−5^ Ω m. They then successfully created metal-semiconductor nanowires on a 17 nm wide and approximately 420 nm long rod-shaped DNA origami [43,44]. They first attached four DNA-modified 50-nm-long Au nanorods to the DNA origami at 60 nm, 70 nm, and 80 nm gaps with DNA hybridization. Cetyltrimethylammonium bromide (CTAB)-coated Te nanorods were then bound in the gaps through electrostatic interaction. Au-Te-Au junctions prepared after electroless plating of Au nanorods showed current-voltage properties corresponding to Schottky junctions [43]. Au-Te-Au junctions have also been successfully achieved by coating Au nanorods with polybenzimidazole (PBI) without electroless plating, followed by annealing at 170 °C (Figure 7) [44]. They recently attached CdS nanorods together with Au nanorods to similar rod-shaped DNA origami and formed CdS-Au nanoscale Schottky contacts by growing Au nanorods through electroless plating. Tungsten probes were fabricated by EBID, and electrical properties were measured. The nonlinear Schottky barrier behavior, with electrical conductance ranging from 0.5 × 10^−4^ to 1.7 × 10^−4^ S, was observed [45].

Bayrak et al. took a different approach to constructing Au nanowires on DNA origami. They arranged eight DNA-modified Au nanoparticles (AuNPs) of 5 nm in diameter into a C-shape of 70 nm long using DNA hybridization and connected them by electroless gold deposition [46]. Charge transport measurements of the nanowires were performed after gold electrodes were prepared by EBID. Hiller et al., on the other hand, used triangular and V-shaped DNA origami as a core for Ag seeding followed by Au coating by the electroless reduction to obtain gold hollow nanotriangles and V-shaped meta-atoms [47].

**Figure 8:**
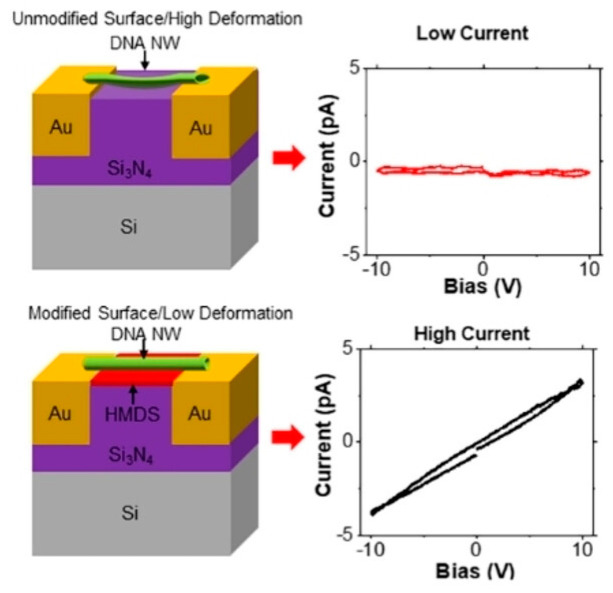
Measurement of electrical conductance of DNA origami nanowires (NW). DNA origami NW bridging Au electrodes separated with Si_3_N_4_ gap significantly deformed, whereas hexamethyldisilazane (HMDS) treatment of the gap suppressed such deformation. Reprinted with permission from [48]. Copyright 2022 John Wiley & Sons.

In contrast to the above studies constructing metal nanowires, Marrs et al. directly measured the electrical conductance of unmodified DNA origami nanowires themselves (Figure 8) [48]. They prepared a 6-helix bundle dimer nanowire (6HB NW) of approximately 800 nm long and a 10-helix bundle pentamer nanowire (10HB NW) of approximately 1.25 µm long. When these NWs were bridged to gold electrodes with 1 µm silicon nitride (Si_3_N_4_) gaps in between, it was found that the height of both NWs decreased by 1 to 2 nm, probably because of electrostatic interaction with the substrate. Treatment of the surface with hexamethyldisilazane (HMDS), on the other hand, significantly suppressed such deformation. By measuring electrical conductivity, they found a difference in resistance of approximately one order of magnitude between the deformed DNA NWs on Si_3_N_4_ (∼10^14^ Ω) and the intact NWs on HMDS (∼10^13^ Ω).

### 3.2. RNA Origami and Metal-Ion Mediated Oligonucleotide Origami

**Figure 9:**
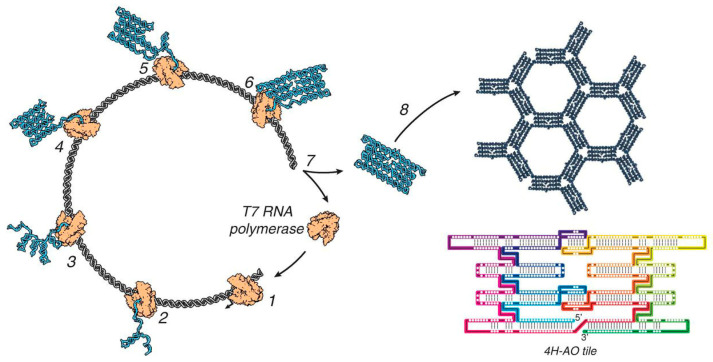
RNA origami. Single-stranded long RNA is transcribed from a DNA template and intramolecularly folds into a designed tile structure (4H-AO tile). The half of the tile is fully synthesized and folded into a domain composed of 11 helical subdomains within steps 1–4. The kissing loops then connect the second half after the synthesis in steps 5 and 6. Released tiles self-assemble into lattices (steps 7 and 8). From [49]. Reprinted with permission from AAAS.

Like the relationship between peptide origami and protein origami, oligonucleotide origami for DNA origami is also conceivable, but oligonucleotides themselves are capable of spontaneous folding without metal ions through complementary base pairing. RNA origami method (Figure 9), which does not require staple strands but allows long single RNA strands to fold into desired structures through intramolecular self-complementary sequences, indeed takes advantage of this property [49,50,51,52,53].

**Figure 10:**
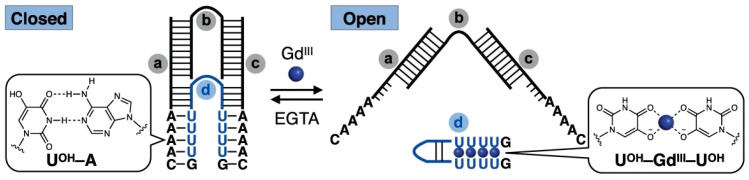
Gd(III) mediated mechanical control of DNA tweezers [56]. Strand b connects strands a and b to construct the DNA tweezers, and closing strand d modified with multiple U^OH^ residues forms intramolecular hairpin in the presence of Gd(III) and open up the DNA tweezers.

On the other hand, several metal ion-mediated non-Watson–Crick base pairings are known, and metal ion-dependent DNA double-strand structures have been studied mainly by Japanese researchers. Ono et al. pursued the interaction between natural nucleobases and metal ions and reported various non-Watson-Crick base pairs such as dT-Hg(II)-T and C-Ag(I)-C, as well as many metal-ion-dependent duplex formation systems utilizing them [54]. Shionoya et al., on the other hand, have synthesized a variety of non-natural nucleobases that coordinate to metal ions and realized Cu(II)- and Hg(I)-mediated double-strand formation [55]. Recently, they have succeeded in controlling toehold-mediated strand displacement, a basic technology in DNA computing, mechanical movement of DNA tweezers (Figure 10), and DNAzyme in a Gd(III)-dependent fashion by using U^OH^-Gd(III)-U^OH^ non-Watson-Crick base pairs [56].

## 4. Biosensors

Biosensors consist of molecular recognition sites and transducers. For the former, as already described, synthetic molecules such as DNA aptamers and peptamers [57,58,59] have been used instead of biomolecules, such as antibodies or enzymes. For the latter, transducers use either or both changes in the weight, dielectric constant, impedance, etc., associated with the molecular recognition sites coupling with target molecules. In this section, we discuss the advantages and disadvantages of the transducers and methods to improve them by using nanostructures.

### 4.1. Detection of Weight Change: Quartz Crystal Microbalance (QCM)

QCM is well known as one of the highly sensitive transducers. The sensing mechanism is that the change in the resonant frequency of a quartz crystal is proportional to the weight of the material attached to the electrode surface. This relationship is well known as the Sauerbrey equation [60]. In addition, quartz crystal is an inexpensive material, and QCM is relatively easy to measure. We can measure viscoelastic changes as well as mass changes in adhered materials by using QCM. It is called QCM-D, here, D stands for dissipation. Since sequential analysis is also possible by connecting the device to a fluid system, QCM with flow injection analysis (FIA) is reported in many studies [61,62]. Generally, QCM is affected by the external environment, such as temperature and humidity; therefore, noise rejection is an issue. To overcome this problem, twin-sensor QCM is developed by Nihon Dempa Kogyo, Co., Ltd. (Tokyo, Japan), and we have reported an actual example [63]. In this case, two measurement areas (electrodes) are provided on a single quartz crystal. The molecular recognition molecule is immobilized in one area, and the other area is covered with materials to avoid nonspecific adsorption and to eliminate environmental noise. In QCM measurements, the resonant frequency is inversely proportional to the thickness of the crystal, which is reaching its limit as the needs for analysis increase in sensitivity, such as sensing the order of pg. Among commercially available products, the resonant frequency of 30 MHz is the most sensitive, and its thickness is approximately 55 µm. Therefore, it is very easy to break and difficult to handle, but its sensitivity is reached to 0.02 ng/Hz in the catalog value.

**Figure 11:**
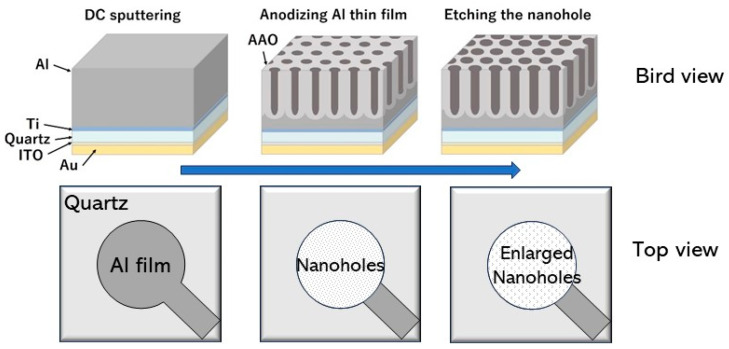
Schematic representations of the fabricating process of QCM-based sensors with anodized aluminum oxide (AAO) nanostructures [64]. DC sputtering, direct current sputtering; ITO, indium-tin-oxide.

To increase the sensitivity, we fabricated nanolevel through-holes by anodizing an aluminum thin film that was coated on one side of the quartz crystal shown in Figure 11. This device would provide the transducer with more molecular recognition sites because of the increase in specific surface area. When antigen-antibody reactions were measured using this device, the sensitivity was successfully improved by a factor of three compared to flat quartz crystals [64]. The results show that our device has the potential to improve the sensitivity of QCM on the use of artificial synthetic materials such as aptamers [65,66,67].

### 4.2. Detection of Dielectric Constant Change: SPR or LSPR

Surface plasmon resonance (SPR) needs a metallic thin film formed on a prism. When light is irradiated to the prism under total reflection conditions, plasmon resonance occurs, in which light resonates with free electrons in the metallic film. At this condition, a sharp drop in reflectance occurs at a certain angle of incidence. This angle is called the plasmon resonance angle and is very sensitive to changes in the refractive index, in other words, the dielectric constant, of the thin film surface [68,69]. To measure the plasmon resonance angle, it needs to adjust the angle of incident light using a goniometer. As a result, the SPR sensing system becomes larger and more expensive. On the other hand, a system for localized surface plasmon resonance (LSPR) is quite simple. LSPR uses metal nanoparticles. When light is irradiated onto metal nanoparticles of several to several tens of nanometers, the free electrons of the metal particles and the light resonate at a specific wavelength [70,71]. This is a simple mechanism of LSPR. In this case, light is incident perpendicular to the device surface, and the reflection or absorption spectrum is measured. When the target materials adhere to the surface of metallic nanoparticles, the dielectric constant of the surface changes, which causes the redshift of the spectrum.

We have developed an LSPR sensing device that combines light interference and LSPR using anodic alumina oxide (AAO) with high aspect ratio nanoholes and have reported an example of its actual use in antigen-antibody reactions [70]. The results make it possible to apply the device to artificial synthetic materials such as DNA aptamers. However, the provided process is somewhat complex.

**Figure 12:**
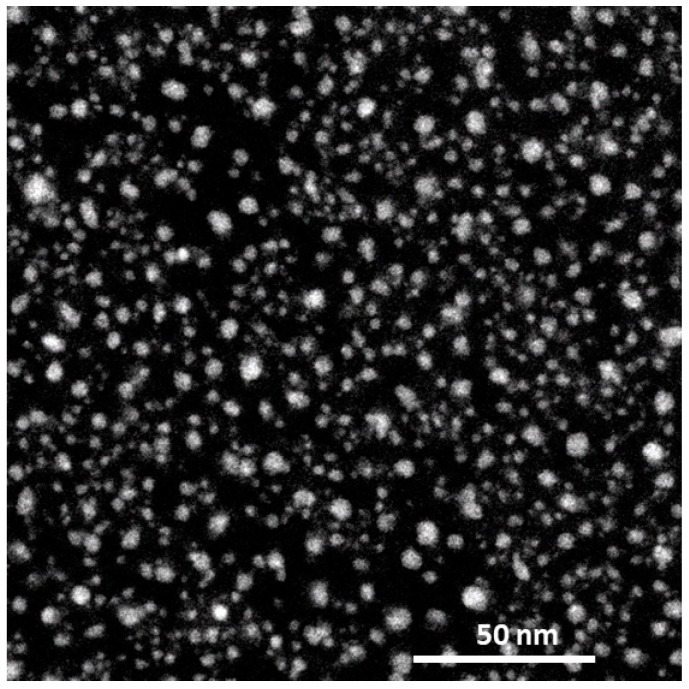
TEM image of Fe nanoparticles with the applied voltage of 90 V and 2200 µF (*n* = 20). Nanoparticles with a diameter of approximately 3.3 nm were deposited with good dispersion.

To fabricate metal nanoparticles with high precision, an EB lithography system [71] and agglomerating nanoparticles by heat treatment after forming a thin film [72] have been reported. The former is difficult to apply to industry use due to the enormous time and cost, and the latter makes it difficult to control the size of the nanoparticles. Therefore, we focus on arc plasma deposition (APD), which is a dry process and does not require any post-treatment steps. APD has been applied to prepare fuel cell catalysts [73] and electrochemical detection electrodes [74] but has never been applied to LSPR. We prepare Fe and Au nanoparticles using APD for LSPR. APD can form nanoparticles by adjusting the parameters of capacitance (C), number of pulses (n), applied voltage (V), deposition pressure, and temperature during deposition. For example, Figure 12 shows a TEM image of Fe nanoparticles, which shows nanoparticles with a diameter of approximately 3.3 nm were deposited with good dispersion. We are currently developing a device for LSPR using gold nanoparticles deposited by APD.

### 4.3. Impedance Change: Electrochemical Impedance Spectroscopy (EIS)

**Figure 13:**
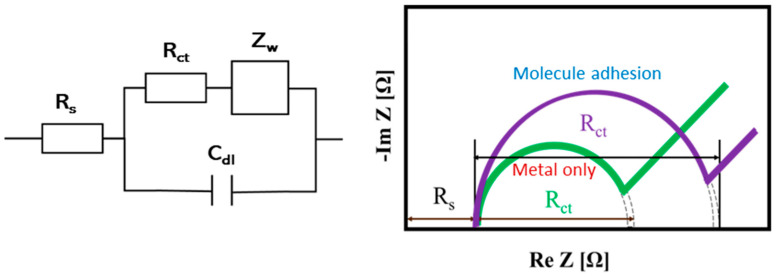
Left: The equivalent circuit for electrochemical measurement. R_s_, resistance of the electrolyte; R_ct_, charge transfer resistance; Z_w_, mass transfer process (diffusion); C_dl_, electrical double layer capacitance. Right: Examples of Nyquist plots before and after molecular adhesion.

Electrochemistry is very famous as a transducer. Electrochemical measurements utilize a special measuring device called a potentiostat. In recent years, the potentiostat has become smaller and can be attached to palmtop devices or smartphones [75], which makes them excellent matches for biosensors. Amperometry has been the main technique for electrochemical measurements in the past, but in recent years, impedance measurement (EIS) has been used since it is highly sensitive to changes in electrode surfaces [76,77]. In electrochemical measurements, the electrochemically active substance (mediator) is needed, and the charge transfer resistance (R_ct_) and mass transfer process (diffusion) (Z_w_) work as impedance. In addition, an electrical double layer is formed at the electrode interface and works as capacitance (C_dl_). There is a resistance of the electrolyte, R_s_, by nature. The equivalent circuit for electrochemical measurement is shown on the left in Figure 13. When we measure the impedance of this circuit while changing the frequency of the applied voltage, we obtain Nyquist plots shown right in Figure 13. The diameter of the semicircle on the Nyquist plots corresponds to R_ct_. In biosensing, we focus on changes in R_ct_. The R_ct_ is small when we measure metal electrodes only because the exchange of electrons with the mediator is smooth. However, when recognition sites such as aptamers are immobilized on the electrode, the R_ct_ increases because the exchange of electrons is hindered by aptamers. Furthermore, the resistance increases when the molecule of interest binds. We have reported an example of using EIS to measure the adsorption and rupture processes of liposomes [78]. There are countless possible combinations of such measurement systems and aptamers [79,80,81,82,83], most of which are expected to be explored. This technique would attract more and more attention in the future.

### 4.4. DNA Origami for Biosensing

Among molecular origami, DNA origami was the first to be used in electrochemical/biosensing studies, which began to be reported in the 2020s. Linko and Corrigan et al. successfully constructed electrochemical DNA (e-DNA) biosensors utilizing DNA origami tiles in combination with electrodes [84]. They prepared a rectangular, peg-board like DNA origami tile, which can be decorated with six single-stranded DNAs on each side. The strands act as binding sites of the target 115-nt strand, which bridges the binding of the DNA origami tile to the probes functionalized on the polycrystalline gold electrode (PGE) to create a sandwich assay boosting R_ct_ at target binding. The authors concluded that DNA origami amplification enhances the limit of detection (LoD) by three orders of magnitude compared to conventional probe DNA design (8.86 pM vs. 3.22 nM).

Kaminska and Tinnfeld et al. took different approaches to construct biosensors with DNA origami [85]. They utilized graphene in graphene-on-glass coverslips as a broadband and unbleachable energy-transfer acceptor without labeling. Three types of DNA origami nanostructures (pillar-shaped, L-shaped, and cubic) were attached to graphene by pyrene modifications and used as the platforms to determine the height of molecules with respect to graphene or the orientation of fluorescence resonance energy transfer (FRET) pairs. Real-time monitoring of graphene energy transfer (GET) also enabled visualization of the dynamics of DNA nanostructures.

A unique application of DNA origami in microbial fuel cells (MFC) has been reported by Wang et al. [86]. Triangular DNA origami was used as a carrier for methylene blue (MB), a good mediator for MFC, as well as a strong DNA binder through intercalation. Nearly 60% of 100 µM MB was loaded onto 5 nM DNA origami, which was then attached to carbon felt to serve as the anode. The authors estimate 36% higher power densities with DNA carriers compared to MB-modified electrodes.

## 5. Conclusions and Perspectives

There is growing interest in the development of functional molecules using amino acids and nucleic acids as building blocks. In particular, innovative approaches such as introducing artificial motifs that cannot be found in natural biomolecules, utilizing complex formations with metal ions, and combining nucleic acids with amino acids are expected to pave the way for creating artificial molecules. The condensation and linkage of individual building blocks to synthesize a single chain have become relatively straightforward tasks. Therefore, it is not difficult to envision that the development of techniques to fold these single chains into desired structures will lead to the creation of novel molecular groups that can be described as “molecular origami”. In contrast to DNA origami, which can handle several thousands of nucleotides to create submicron subunits and even micron-scale assemblies, protein origami can still treat fewer amino acids. The rapid development of artificial intelligence for protein structure predictions [87], however, should aid and accelerate the advancement of de novo design of much larger protein origami, in combination with nucleic acids origami, in the coming years.

As discussed in this article, the combination of peptides containing non-natural amino acids with metal ions, as well as the fusion of DNA origami with proteins/peptides and their integration with metal complexes, may enable the construction of novel molecular origami structures. The creation of such molecular groups could facilitate advancements in various fields, including the development of drug delivery systems and biosensors in medicine, new nanomaterials and biomaterials in material science, innovative catalysts such as artificial enzymes, and even of “Molecular Robots” and “Chemical Artificial Intelligence” [88,89]. Exciting new developments in this field are highly anticipated.
